# Clinical Characteristics and Predictors of Outcome for Onconeural Antibody-Associated Disorders: A Retrospective Analysis

**DOI:** 10.3389/fneur.2017.00584

**Published:** 2017-11-13

**Authors:** Shaohua Liao, Ying Qian, Huaiqiang Hu, Bing Niu, Hongwei Guo, Xiaoling Wang, Shuai Miao, Chuanfen Li, Bingzhen Cao

**Affiliations:** ^1^Department of Neurology, Graduate School of the Second Military Medical University, Shanghai, China; ^2^Department of Neurology, General Hospital of Jinan Military Command of PLA, Jinan, China; ^3^Department of Neurology, People’s Liberation Army 152 Hospital, Pingdingshan, China

**Keywords:** onconeural antibody, clinical characteristics, management, outcome, predictors

## Abstract

**Objective:**

To describe and analyze the clinical characteristics, laboratory data, management, and outcome of patients with onconeural antibody-associated disorders (OAAD) and identify predictors for poor outcome.

**Methods:**

This was a retrospective review of all patients with potential OAAD, who were hospitalized in Jinan General Hospital between September 2009 and July 2017. We clarified the diagnosis, collected comprehensive information and categorized patients into three groups: paraneoplastic neurological disorders (PNDs), autoimmune encephalitis (AE), and possible OAAD. Within the three groups, we analyzed a range of clinical and laboratory parameters and used univariate and multivariate regression analysis to identify predictors for poor outcome [modified Rankin Scale (mRS) = 3–6].

**Results:**

From 158 patients, we identified 70 who fulfilled the criteria for OAAD, including 44 men (62.9%) and 26 women (37.1%). There were 38 patients (54.3%) in the PNDs group, 14 patients (20%) in the AE group, and 18 patients (25.7%) in the possible OAAD group. After the last follow-up, 14 (36.8%), 9 (64.2%), and 12 (66.7%) had a good outcome (mRS = 0–2). However, 6 (15.8%), 2 (14.3%), and 3 (16.7%) died, respectively. Univariate analysis showed that duration prior to the hospital (*p* = 0.0224) and urinary incontinence/retention (*p* = 0.0043) were associated with poor outcome (mRS = 3–6). After multivariate regression analysis, urinary incontinence/retention (*p* = 0.0388) and an immunocompromised state (*p* = 0.0247) remained as significant factors for poor outcome.

**Conclusion:**

Urinary incontinence/retention and an immunocompromised state represent significant predictors of a worse prognosis for patients with OAAD. By contrast, cerebrospinal fluid analysis showed that serum autoantibodies and tumor markers, the function of crucial organs, electrophysiology, and radiological findings were not associated with a poor outcome.

## Introduction

Onconeural antibody-associated disorders (OAADs) are a type of heteroplasmic neurological syndrome and are drawing increasing attention from neurologists. OAADs are usually associated with some form of neuronal autoantibody, part of which is known to be associated with systemic cancer. The target antigens include nuclear or cytoplasmic proteins, such as Hu, Yo, Ri, or intracellular synaptic proteins, such as 65 kDa glutamic acid decarboxylase, amphiphysin, or cell-surface or synaptic proteins, such as the *N*-methyl-d-aspartate receptor (NMDAR), the α-amino-3-hydroxy-5 -methyl-4-isoxazolepropionic acid receptor, and the γ-aminobutyric acid receptor-B (GABABR) (1–3). Furthermore, OAADs can be broadly divided into two clinical categories: classic paraneoplastic neurological syndromes (PNDs) and autoimmune encephalitis (AE) (4).

Paraneoplastic neurological disorders are mostly accepted as neurological syndromes that are immune mediated and triggered when systemic tumors express certain neuronal antigens (3). PND syndromes can manifest in the central nervous system, peripheral system, neuromuscular junction and muscle (5). However, AE generally refers to a group of disease processes that share analogical clinical features and neuroimaging findings and are largely differentiated by characteristic antibodies which mediate immune attacks on different neural structures (6). The presentations of AE include memory or behavioral deficits, new focal findings, seizures, cerebrospinal fluid (CSF) pleocytosis, and abnormalities upon magnetic resonance imaging (MRI) (7). Previous reports have indicated that approximately 1 in 10,000 patients with cancer eventually develop PNDs (8), thus showing that this disease is associated with a low prevalence. In other research, patients were preselected using clinical criteria and serological screening; these data showed that 163/649 (25%) cases were serologically positive for antibodies associated with PNDs during a 23-month period (4). In terms of AE, the annual incidence of encephalitis, of any etiology, is 2–3 cases per 100,000 patients in northern Europe (9), of which, at least 20% are immune mediated. The predominant cohort of patients for AE includes those with anti-NMDA-receptor encephalitis (4%) and voltage-gated potassium channels (VGKC)-complex antibody positive encephalitis (3%) (9).

In the present study, we analyzed the medical records from a series of patients with OAAD, investigated the spectrum of clinical characteristics and management strategies associated with this disease and evaluated potential predictors which could be used to influence outcome.

## Materials and Methods

### Standard Protocol Approvals and Patient Consent

The study was approved by the ethics committee of the General Hospital of Jinan Military Command (NO. 201611). All patients provided informed written consent to allow their medical records to be used in this study. The study protocol conformed to the ethical guidelines of the 1975 Declaration of Helsinki.

### Study Design

We conducted a retrospective review of all patients with potential PNDs or AE between September 2009 and July 2017, and who were tested for classical paraneoplastic antibodies and autoimmune synaptic antibodies. Records were manually reviewed and patients with a final diagnosis of PNDs, AE, or possible OAAD were recruited into this study.

### Definitions Used in This Study

We used specific diagnostic criteria to define PNDs and AE (5). Definite PNDs were defined as follows: (1) a classical syndrome and cancer that develops within 5 years of the diagnosis of neurological disorder; (2) a non-classical syndrome that resolves or significantly improves after cancer treatment without concomitant immunotherapy, provided that the syndrome was not susceptible to spontaneous remission; (3) a non-classical syndrome with onconeural antibodies (well characterized or not) and cancer that develops within 5 years of the diagnosis of neurological disorder; (4) A neurological syndrome (classical or not) with well characterized onconeural antibodies (anti-Hu, Yo, CV2, Ri, Ma2, or amphiphysin), but without cancer. Possible PNDs were defined as described previously (5): (1) a classical syndrome, no onconeural antibodies, no cancer but at high risk of an underlying tumor; (2) a neurological syndrome (classical or not) with partially characterized onconeural antibodies but without cancer; and (3) a non-classical syndrome, no onconeural antibodies, and the presence of cancer within 2 years of diagnosis.

Definite AE was defined, as described previously (7): (1) sub-acute onset (rapid progression of less than 3 months) of working memory deficits (short-term memory loss), altered mental status, or psychiatric symptoms; (2) at least one of the following: new focal CNS findings; seizures which could not be explained by a previously known seizure disorder; CSF pleocytosis (white blood cell count of more than five cells per mm^3^); MRI features suggestive of encephalitis; (3) positive antibody testing in the serum or CSF, mainly targeting cell-surface or synaptic proteins; and (4) reasonable exclusion of other disorders. Possible AE was defined as an absence of positive antibody testing in the serum or CSF.

The “duration prior to the hospital” was defined as the number of days from the onset of symptoms to hospitalization in our hospital. A “good outcome” was defined as a grade of 0–2 on the modified Rankin Scale (mRS), while a “poor outcome” was defined as a mRS of 3–6. An “immunocompromised state” was defined as when patients received chemotherapy, chronic immunosuppressants or steroids (for >3 months). IgG index was calculated according to the following formula: [CSF IgG (mg/mL)/serum IgG (mg/mL)]/[CSF albumin (mg/mL)/serum albumin (mg/mL)]. MRI fluid-attenuated inversion recovery (FLAIR)/T2 abnormality was defined as high signal intensity in images of the brain parenchyma. “Total tumors” refers to the total number of tumors for a specific patient, including those which were newly identified, and those recorded in historical records.

### Inclusion/Exclusion Criteria

Patients were included in the study if they concurred with criteria outlined above, and were under treatment in our hospital. We excluded cases if there was any evidence of infectious or post-infectious encephalitis, autoimmune disease-associated encephalitis, toxic-metabolic encephalopathy, brain tumor or metastasis, vitamin deficiency or alcohol-related encephalopathy, side-effects of drugs (chemotherapeutics or others), primary angiitis of the CNS, or other diseases (e.g., acquired immune deficiency syndrome and Creutzfeldt–Jakob disease).

### Categorization of Patients

Patients were categorized into three groups: those with PNDs, AE, or possible OAAD. Patients were categorized as definite PNDs or AE when they concurred with the appropriate definitions. Patients who were classified as possible PNDs or AE were all categorized into the possible OAAD group.

### Clinical Characteristics and Investigations

Basic demographic information (age, gender) were collated for each patient, along with the duration prior to the hospital, and whether there was an immunocompromised state or not. All patients underwent consultation and a neurological examination by an attending neurologist, who ascertained clinical symptoms, neurological signs, and mRS upon admission. The same information was collected just prior to discharge. mRS data were collected by telephone follow-up on a semi-annual basis. During the period of hospital care, intensive care unit (ICU) admission, mechanical ventilation, and therapy were recorded.

In this study, we analyzed a range of laboratory data, including CSF analysis (glucose, protein, white blood cells, IgG index), serum autoantibodies [anti-nuclear antibodies (ANA), onconeural antibodies] and tumor markers [neuron-specific endase (NSE), carcinoembryonic antigen (CEA)]. Data were also acquired so that we could evaluate the function of crucial organs, such as the heart, liver, and kidney and also the relative state of hemopoiesis. Such data included creatine phosphokinase isoenzyme (CK-MB), glutamic oxalacetic transaminase/glutamic-pyruvic transaminase (AST/ALT), urea nitrogen/creatinine (BUN/Cr), the counts of leukocyte, polymorphonuclear and thrombocyte. Electroencephalograms (EEGs) and electromyography (EMG) were also carried out in order to assess electrophysiology. Neuroimaging examinations, including brain MRI FLAIR/T2 abnormality and computed tomography (CT), were also used to investigate for underlying tumors. All of the results were studied from the first time in our hospital.

### Statistics

The data were analyzed using Excel 2016. Normality was assessed using the Shapiro–Wilk Normality Test on R software. Continuous variables, which were normally distributed, were compared between groups with the Student’s *t*-test. For continuous variables that did not follow normal distributions, we used the Mann–Whitney *U* test (Wilcoxon rank-sum test). The Chi-square test or Fisher’s exact test, as appropriate, was used to compare categorical variables between groups, and a binomial logistic regression model was used to evaluate the association between variables and outcome. All statistical analyses were performed in R (the R Project: http://www.R-project.org). Odds ratios (ORs) and their 95% confidence intervals (CIs) were also calculated. The predefined level of significance was 0.05.

## Results

### Demographics and Clinical Characteristics

From a total of 158 patients, we identified 70 patients who fulfilled the criteria for OAAD, including 44 men (62.9%) and 26 women (37.1%) with a median age of 55 years (range: 14–78 years). The median duration prior to the hospital was 74 (range: 7–1,090). The median number of days in hospital was 37 (range: 3–116). A summary of the patient demographics and characteristics is given in Table 1.

**Table 1:** Demographics, clinical presentation, management, and outcome.

Age, sex	Diagnosis	Clinical presentation	Types of onset	Onconeural antibody	Associated tumor	Tumor marker	Coexistent autoantibody	Modified Rankin Scale (mRS) on admission	Therapy	Hospital stay	mRS final
31, M	Anti-NMDAR encephalitis	Double vision	Chronic	*N*-methyl-d-aspartate receptor (NMDAR), Ta			Anti-AQP4, anti-MPO, anti-PR3	3	Steroids	37	2
55, F	Limbic encephalitis	Mental and behavior disorder, cognitive damage, epilepsy	Sub-acute	Amphiphysin	Lung cancer	NSE, CA125		5	steroids, IVIg	17	5
61, F	Peripheral neuropathy	Extremities numbness and weakness	Sub-acute	Hu	Breast cancer, malignant fibrous histiocytoma			4		9	3
37, M	Peripheral neuropathy	Extremities numbness	Chronic	Hu	Lung cancer	NSE, CEA		2		10	2
17, M	Paraneoplastic cerebellar degeneration	Limb seizures	Chronic	SOX1				3		11	3
56, F	Anti-NMDAR encephalitis	Hypomnesis, mental and behavior disorder, confusion	Acute	NMDAR	Small cell lung cancer (SCLC)			5	Steroids, IVIg	47	6
62, F	Peripheral neuropathy	Weakness of limbs	Chronic	CV2, Hu	Lung cancer	NSE, CEA, CA125, CA199		3		7	3
66, M	Paraneoplastic cerebellar degeneration	Barylalia, walk unsteadily	Sub-acute	CV2			ANA	2		17	2
46, F	Encephalomyelitis	Weakness of limbs	Chronic	Yo	Breast cancer, multiple myeloma			2	IVIg	17	6
48, F	Peripheral neuropathy	Extremities numbness and weakness	Chronic	Yo				3		8	6
35, M	Anti-NMDAR encephalitis	Mental and behavior disorder	Acute	NMDAR			Anti-nuclear, anti-M2	3	IVIg, steroids	38	0
72, M	Peripheral neuropathy	Lower limbs weakness	Chronic		Squamonus cell carcinoma of lung	NSE		3		53	6
54, M	Paraneoplastic cerebellar degeneration	Dysarthria, walk unsteadily	Chronic	Ri	Colon cancer			4	Colon surgery	8	4
60, M	Peripheral neuropathy	Waist pain, ache of right lower limb	Sub-acute		Multiple myeloma			4	IVIg, chemotherapy, steroids	116	5
66, M	Gabab encephalitis	Epilepsy, right hemidysesthesia, lower limbs weakness	Chronic	GABAb				3	IVIg	11	2
54, F	Anti-NMDAR encephalitis	unresponsive, mental and behavior disorder	Acute	NMDAR				3	IVIg, steroids	14	2
54, M	Limbic encephalitis	Hypomnesis, unresponsive, epilepsy	Sub-acute			AFP		4	IVIg	15	1
24, F	Anti-NMDAR encephalitis	Headache, hallucination, epilepsy	Acute	NMDAR		CA125		5	IVIg	55	0
52, F	Paraneoplastic cerebellar degeneration	Dizziness, walk unsteadily, nausea and vomit	Sub-acute	Hu	SCLC	CEA, CA125		4	Lung cancer resection	42	2
22, M	Necrotizing myopathy	Weakness of limbs	Sub-acute	Yo		NSE	Anti-Jo1, anti-Mi2, anti-SRP	3	Steroids	23	2
70, M	Limbic encephalitis	Epilepsy	Acute		Rectal cancer	NSE	ANA	2	Steroids, IVIg	47	1
76, M	Myasthenic syndrome	Lower limbs weakness	Chronic	Ta	SCLC	CEA, CA199, CY211		2		5	1
27, M	Anti-NMDAR encephalitis	Fever, epilepsy	Sub-acute	NMDAR		NSE		5	Plasma exchange, cyclophosphamide, IVIg	71	4
76, F	Brainstem encephalitis	Weakness of limbs, dysphagia	Sub-acute	Hu		CEA, CA125, CY211		5	IVIg, steroids	79	6
65, M	Lambert–Eaton myasthenic syndrome	Weakness of limbs	Sub-acute	Yo	Adenocarcinoma of anal canal		Anti-VGCC	4	IVIg, excision of anal canal carcinoma	13	3
37, M	Anti-NMDAR encephalitis	cognitive decline	Chronic	NMDAR		AFP		4	IVIg	4	3
74, F	Motor neuron disease, limbic encephalitis	Barylalia, bucking	Chronic	Yo		NSE		3		4	6
62, M	Peripheral neuropathy	Extremities numbness and weakness	Chronic	Hu		CEA	Anti-SSA, anti-Ro52, anti-HI	4	IVIg, steroids	8	6
72, M	Guillain–Barre syndrome	Extremities numbness and weakness, hoarseness	Acute	Yo		NSE, CEA		2		3	0
14, F	Anti-NMDAR encephalitis	Mental and behavior disorder, epilepsy	Acute	NMDAR			ANA	3		21	0
41, M	Anti-NMDAR encephalitis	Mental and behavior disorder	Acute	NMDAR				3	IVIg	35	1
43, F	Myasthenia gravis	Blepharoptosis, barylalia, dysphagia	Sub-acute	Titin			Anti-GM1, anti-AchR	2		6	1
56, F	Limbic encephalitis	Mental and behavior disorder	Chronic	Hu				4		7	3
40, F	Anti-NMDAR encephalitis	Mental and behavior disorder	Acute	NMDAR				3	Steroids, IVIg	16	1
64, M	Lambert–Eaton myasthenic syndrome	Weakness of limbs	Chronic		SCLC			3		8	3
52, M	Lambert–Eaton myasthenic syndrome	Lower limbs weakness	Chronic		Thymoma			1		7	1
71, M	Peripheral neuropathy+paraneoplastic cerebellar degeneration	Lower limbs weakness	Chronic	Ri	SCLC			3		6	2
63, F	Limbic encephalitis	Headache, mental and behavior disorder, epilepsy	Acute					5	IVIg	16	0
60, M	Chronic Guillain–Barre syndrome	Extremities numbness and weakness	Chronic progressive	Ta		CY211	Anti-GM3, anti-GT1b	3	Steroids	20	0
70, F	Limbic encephalitis	Lower limbs weakness, aconuresis, Hypomnesis, personality change	Chronic	Hu	SCLC	NSE		4		13	3
78, M	Myasthenia gravis	Gatism	Chronic	Hu	Lung cancer	NSE, CA199, CA724, CY211		3		7	3
42, M	Paraneoplastic cerebellar degeneration	Walk unsteadily, involuntary movement	Sub-acute		SCLC		Anti-CB	4	IVIg, cyclophosphamide	40	4
37, M	Lambert–Eaton myasthenic syndrome	Limb weakness, emaciation	Chronic					1		7	1
54, M	Myelitis	Numbness and weakness of the lower limbs	Sub-acute	Hu	Squamous cell lung carcinoma	NSE, CY211		5		11	5
66, F	Motor neuron disease	Labored breathing, hoarseness	Chronic	Hu	Ovary tumor		Anti-AchR	2		5	3
29, F	Paraneoplastic cerebellar degeneration	Walk unsteadily, barylalia	Chronic	Yo	Breast infiltrating ductal carcinoma			2		37	2
56, M	Brainstem encephalitis	Barylalia, lower limbs weakness	Acute	Amphiphysin	SCLC			4	Chemotherapy	16	2
49, M	Vasculitic peripheral neuropathy	Myalgia of the upper legs, intermittent fever	Chronic	Ta		CY211	Anti-Ro52, anti-MPO	3	IVIg, cyclophosphamide	18	2
24, M	Myelitis	Progressive numbness of lower limbs	Sub-acute	Yo				1	Steroids	3	1
54, M	Peripheral neuropathy	Lameness, weakness of the toes	Chronic	CV2	Renal carcinoma			2		9	3
56, M	Motor neuron disease	Weakness of limbs, dysphagia	Chronic		Lung cancer	NSE, CY211		3		7	2
57, M	Lambert–Eaton myasthenic syndrome	Lower limbs weakness, paroxysmal unconsciousness	Chronic		SCLC		ANA	3	Steroids, IVIg	11	2
41, M	Anti-NMDAR encephalitis	Mental and behavior disorder, epilepsy	Acute	NMDAR	Intracranial tumor	NSE	Anti-SSA, anti-Ro52	5	IVIg	90	5
57, F	Paraneoplastic cerebellar degeneration	Dizziness, walk unsteadily	Acute		Lung cancer	NSE		4		10	4
65, F	Myelitis	Numbness and weakness of right lower limb	Sub-acute		Ovarian cancer	CA199	Anti-AQP4	4	Steroids	10	6
49, M	Autoimmune encephalitis	Involuntary movement of limbs, unconsciousness	Acute					2	IVIg, steroids	5	1
63, M	Paraneoplastic cerebellar degeneration	Barylalia, walk unsteadily	Sub-acute		Bladder cancer			3	Steroids	14	2
49, M	Anti-NMDAR encephalitis	Headache	Acute	NMDAR	Adrenal gland neoplasms			1	IVIg	46	6
65, F	Peripheral neuropathy	Progressive numbness and weakness of limbs, bathesthesia	Chronic	Hu			Anti-GM	2		13	5
28, M	Motor neuron disease	Weakness of the upper limbs	Chronic	Yo				2		10	2
77, M	Limbic encephalitis	Mental and behavior disorder	Chronic					4	Steroids, IVIg	9	1
49, M	Paraneoplastic cerebellar degeneration, Lambert–Eaton myasthenic syndrome	Dizziness, walk unsteadily	Chronic		SCLC	NSE		3	Steroids	9	3
60, M	Lambert–Eaton myasthenic syndrome, paraneoplastic cerebellar degeneration	Weakness of limbs	Chronic				Anti-Scl70, ANA	4	Steroids	7	6
44, F	Paraneoplastic cerebellar degeneration	Headache, dizziness, walk unsteadily	Chronic	YO				4		6	3
55, M	Lambert–Eaton myasthenic syndrome	Lower limbs weakness	Acute		Lung adenocarcinoma			2	Lung cancer resection	21	2
61, F	Peripheral neuropathy	Weakness of both hands	Chronic	Hu				4		14	2
70, F	Anti-NMDAR encephalitis	Increases in sleep, lower limbs weakness	Sub-acute	NMDAR	Ovarian teratoma			3	Steroids, IVIg	27	2
40, F	Encephalomyelitis	Lower limbs weakness, hypopsia	Chronic	Am	Breast infiltrating ductal carcinoma			4		11	4
43, M	Myelitis	Pain of right waist, numbness and weakness of lower limbs	Chronic	Ta				3	Steroids, azathioprine	21	3
69, M	Motor neuron disease	Barylalia, dysphagia, weakness of the upper limbs	Chronic	Yo				3		7	6

*NSE, Neuron-specific endase; AFP, alpha fetoprotein; CEA, carcinoembryonic antigen; CA, carbohydrate antigen; CY, cytokeratin; AQP, Aquaporin; MPO, myeloperoxidase; PR, protease; ANA, anti-nuclear antibodies; M2, mitochondrion 2; Jo1, histidyl tRNA synthase 1; Mi2, 218/240 kDa helicase family protein; SRP, signal recognition particle; VGCC, Calcium ion channel antibody; SSA, Sjogren syndrome A; Ro -52, 52k Da Sjogren syndrome A; HI, histone; GM, gangliosides; AchR, acetylcholine receptors; GT1b, trisialic acid ganglion; CB, centromere protein B; Scl, scleroderma; IVIg, intravenous immunoglobulin*.

In this study, 38 patients (54.3%) were classified into the PNDs group, including 17 women (44.7%) and 21 men (55.3%). There were 14 patients (20%) diagnosed with AE, including 6 women (42.9%) and 8 men (57.1%). The other 18 patients (25.7%) were classified as having possible OAAD, including 3 women (16.7%) and 15 men (83.3%). There were 17 patients (24.3%) with mental and behavioral abnormalities, 13 (18.6%) with epilepsy, and 12 (17.1%) with autonomic neuropathic lesions. Detailed statistical analysis of patient demographics, clinical presentation, and hospital course, in each of the three groups, is given in Table 2.

**Table 2:** Demographics, clinical presentation, and management.

	Paraneoplastic neurological disorders (*n* = 38)	Autoimmune encephalitis (*n* = 14)	Possible onconeural antibody associated disorders (*n* = 18)
Age, year	55.5 (22–78)	40.5 (14–70)	60 (17–77)
Male	21 (55.3%)	8 (57.1%)	15 (83.3%)
**Before hospitalization**			
Immunocompromised state	9 (23.7%)	3 (21.4%)	5 (27.8%)
Duration prior to the hospital, days	120 (15–1,090)	22.5 (8–365)	60 (7–365)
**Clinical presentation**			
Cognition damage	4 (10.5%)	10 (71.4%)	3 (16.7%)
Psychological and behavioral abnormality	4 (10.5%)	10 (71.4%)	3 (16.7%)
Confusion	1 (2.6%)	5 (35.7%)	1 (5.6%)
Epilepsy	2 (5.3%)	7 (50%)	4 (22.2%)
Focal deficits	21 (55.3%)	1 (7.1%)	8 (44.4%)
Urinary incontinence/retention	6 (15.8%)	3 (21.4%)	3 (16.7%)
**During hospitalization**			
Temperature upon admission, °C	36.5 (35.9–37.1)	36.55 (36.2–39)	36.4 (36–37.8)
ICU admission	1 (2.6%)	4 (28.6%)	1 (5.6%)
Mechanical ventilation	1 (2.6%)	4 (28.6%)	0
Immunotherapy	11 (28.9%)	13 (92.9%)	11 (61.1%)
Total tumors	23 (60.5%)	4 (28.6%)	8 (44.4%)
Hospital stay, d	10 (3–79)	36 (4–90)	10.5 (3–116)

*Data presented as *n* (%) or median (range)*.

*ICU, intensive care unit; WBC, white blood cell; ADA, adenosine deaminase; Glu, glucose; ALB, albumin; QALB, albumin quotient; TGAb, antithyroglobulin antibody; TPOAb, anti-thyroid peroxidase antibody; NSE, Neuron-specific endase; AFP, alpha fetoprotein; CEA, carcinoembryonic antigen; CA, carbohydrate antigen; CY, cytokeratin; CK-MB, creatine phosphokinase isoenzyme; AST, glutamic oxalacetic transaminase; ALT, glutamic-pyruvic transaminase; BUN, urea nitrogen; Cr, creatinine; PMN, polymorphonuclear; EEG, electroencephalogram; EMG, electromyography; FLAIR, fluid-attenuated inversion recovery*.

### Investigations

During hospitalization, 60 patients (85.7%) underwent CNS examination by MRI and 18 patients (30%) were identified as having FLAIR/T2 abnormalities. Furthermore, 27 patients (38.6%) received EEG examinations, 19 of which (70.4%) showed an abnormal slow wave or a sharp and slow wave. EMG was performed in 41 patients (58.6%) and identified 37 positive cases (90.2%). Selected patients underwent CSF examination to determine cell count, protein concentration, and immunoglobulin (Ig) concentration. We also tested characterized onconeural antibodies (anti-Hu, Yo, Ri, Ma2, CV2, and amphiphysin) in 67 patients (95.7%) and NMDAR antibody in 27 patients (38.6%). A further cohort of 59 patients (84.3%) were screened for serum tumor markers, including NSE, AFP, CEA, CA125, CA153, CA199, CA724, and CY211; 26 positive cases (44.1%) were identified. Coexistent autoantibodies were also examined in 37 patients (54.3%), including anti-nuclear/myositis/ganglioside antibody profiles, anti-myeloperoxidase (MPO)/protease 3 (PR3)/acetylcholine receptors (AchR)/aquaporin 4 (AQP4) antibodies, and 19 patients (51.4%) were shown to exhibit the coexistence of other autoantibodies.

We also collected a range of other data to allow us to evaluate the status of primary organ function. All patients were tested for AST/ALT, BUN/Cr, the counts of leukocyte, polymorphonuclear, and thrombocyte, and CK-MB was tested in 44 patients (62.9%). The results were analyzed to identify potential predictors of outcome and are presented in Table 3.

**Table 3:** Cerebrospinal fluid (CSF), other test results, electrophysiology, and radiological findings.

	Paraneoplastic neurological disorder	Autoimmune encephalitis	Possible
**CSF analysis**			
WBC, ×10^6^/L	9.0 (9.0–60.0), 19	10.5 (0.0–96.0), 12	2.0 (1.0–30.0), 12
Protein, mg/L	0.5 (0.2–1.5), 18	0.5 (0.2–0.9), 12	0.5 (0.2–3.9), 12
Cl, mmol/L	122.2 (107.0–128.3), 17	124.5 (109.3–129.7), 12	121.1 (106.4–132.8), 12
Glu, mmol/L	3.7 (2.5–5.2), 17	3.4 (3.2–4.7), 12	3.6 (3.1–8.6), 12
Qalb, ×1,000	7.6 (3.5–63.8), 11	7.5 (2.6–16.1), 9	9.0 (2.2–70.0), 9
IgG Index, ×1,000	593.3 (231.1–791.9), 7	548.0 (161.9–721.5), 5	503.7 (348.1–701.1), 5
**Serum autoantibody and tumor markers**			
TGAB, IU/mL	0.1 (0.0–38.7), 30	0.2 (0.0–175.0), 10	1.5 (0.1–36.0), 10
TPO, IU/mL	0.4 (0.1–39.7), 30	0.7 (0.1–131.0), 10	1.7 (0.2–1,087.0), 10
Other autoantibodies	9 (50%), 18	4 (44.4%), 9	6 (60%), 10
Neuron-specific endase, μg/L	14.0 (7.0–180.5), 27	11.7 (8.6–30.1), 10	14.5 (9.1–47.9), 10
AFP, ng/mL	2.9 (0.6–6.0), 28	1.3 (0.6–8.5), 11	2.9 (1.5–10.3), 15
Carcinoembryonic antigen, μg/L	2.4 (0.5–13.1), 30	2.3 (1.0–4.5), 11	1.7 (0.5–7.6), 15
**Organic function evaluation**			
CK-MB, U/L	10.0 (4.0–341.0), 26	13.0 (6.0–98.0), 9	11.0 (6.0–24.0), 9
AST/ALT, ×100	110.4 (37.0–666.7), 38	127.7 (32.5–204.8), 14	117.4 (45.6–214.3), 9
BUN/Cr, ×100	6.6 (3.0–13.2), 38	7.5 (1.8–12.6), 14	6.5 (2.0–14.5), 18
WBC, ×10^9^/L	6.1 (2.5–21.7), 38	8.3 (5.3–18.4), 14	7.9 (3.9–11.9), 18
PMN, %	65.8 (35.9–90.1), 38	72.8 (58.5–92.7), 14	73.0 (43.7–94.5), 18
Platelet, ×10^9^/L	229.5 (102.0–587.0), 38	223.0 (140.0–377.0), 14	232.5 (53.0–363.0), 18
**Electrophysiology and radiological findings**			
Electromyography	26 (96.3%), 27	0	8 (72.7%), 11
Electroencephalogram	4 (80%), 5	11 (78.6%), 14	6 (75%), 8
Magnetic resonance imaging FLAIR/T2 abnormalities	10 (33.3%), 30	5 (35.7%), 14	3 (18.8%), 16
Computed tomography finding on tumor	10 (35.7%), 28	1 (8.3%), 12	2 (15.4%), 13

*Data presented as positive specimen (%) sample size, or median (range) sample size*.

### Diagnosis and Treatment

Patients included in this study were divided into three groups: those with PNDs (38 patients, 54.3%), AE (14 patients, 20%) and possible OAAD (18, 25.7%). The main diagnoses were anti-NMDAR encephalitis (13/70 patients, 18.6%), peripheral neuropathy (12/70 patients, 17.1%), paraneoplastic cerebellar degeneration (12/70 patients, 17.1%) and Lambert-Eaton myasthenic syndrome (8/70 patients, 11.4%). The main onconeural antibodies detected were anti-Hu (13/70 patients, 18.6%), anti-NMDAR (13/70 patients, 18.6%), and anti-Yo (11/70 patients, 15.7%). Overall, 36 patients (51.4%) were found to possess associated tumors, the most common tumor being lung cancer (19/36 patients, 52.8%), particularly small cell lung cancer (SCLC) (10/19 patients, 52.6%).

In total, 39 patients (55.7%) underwent anti-tumor or immunotherapy and 4 patients (10.3%) received tumor surgery, 22 patients (56.4%) were administered with steroids, 26 patients (66.7%) were treated with IVIg, and 13 patients (33.3%) received both steroids and IVIg. Some patients were admitted into the ICU (6/70 patients, 8.6%), and five patients (7.1%) required mechanical ventilation.

### Outcomes

At discharge, there were 17 patients (44.7%) with PNDs, 9 (64.3%) with AE and 10 (55.6%) with possible OAAD who had a good outcome. By contrast, there were 21 (55.3%) patients with PNDs, 5 (35.7) with AE and 8 (44.4%) with possible OAAD who had a poor outcome. After the last follow-up (median: 23 months; range: 0–84), 14 patients (36.8%), 9 patients (64.2%), and 12 patients (66.7%) had a good outcome. However, six patients (15.8%), two patients (14.3%), and three patients (16.7%) died in the three cohorts, respectively.

### Predictors of Outcome

After the last follow-up, the entire patient cohort was classified into groups of good (mRS = 0–2) or poor (mRS = 3–6) outcome after the last follow-up. Univariate analysis was then used to assess variables which were potentially associated with the functional outcome of patients (Table 4). Univariate analysis showed that duration prior to the hospital (*p* = 0.0224) and urinary incontinence/retention (*p* = 0.0043) were factors associated with poor outcome.

**Table 4:** Variables associated with functional outcome in onconeural antibody associated disorders patients: univariate analysis.

	Good outcome (*n* = 35)	Poor outcome (*n* = 35)	*p*-Value
Age, year	54 (14–77), 35	56 (17–78), 35	0.1593
Age > 65	8 (22.9%), 35	10 (28.6%), 35	0.7845
Male	25 (71.4%), 35	19 (54.3%), 35	0.2162
Immunocompromised state	5 (14.3%), 35	12 (34.3%), 35	0.0944
Duration prior to the hospital, day	60 (7–365), 35	120 (9–1,090), 35	0.0224
Hospital stay, day	15 (3–55), 35	10 (4–116), 35	0.5215
Cognition damage	10 (28.6%), 35	7 (20.0%), 35	0.5772
Mental and behavior disorder	10 (28.6%), 35	7 (20.0%), 35	0.5772
Confusion	2 (5.7%), 35	5 (14.3%), 35	0.4283
Epilepsy	9 (25.7%), 35	4 (11.4%), 35	0.2189
Focal deficits	12 (34.3%), 35	18 (51.4%), 35	0.2272
Urinary incontinence/retention	1 (2.9%), 35	11 (31.4%), 35	0.0043
Temperature upon admission, c	36.4 (35.9–39), 35	36.5 (36–38.7), 35	0.3327
ICU admission	2 (5.7%), 35	4 (11.4%), 35	0.6733
Mechanical ventilation	1 (2.9%), 35	4 (11.4%), 35	0.3565
Total tumors	15 (42.9%), 35	24 (68.6%), 35	0.0542
Immunotherapy	19 (54.3%), 35	16 (45.7%), 35	0.4621
Cerebrospinal fluid (CSF) WBC, ×10^6^/L	4 (0–26), 21	8.5 (0–96), 22	0.1365
CSF PRO, mg/L	0.5 (0.2–3.9), 21	0.5 (0.3–1.5), 21	0.2627
CSF Cl, mmol/L	123.1 (106.4–132.8), 20	121.4 (107–128.3), 21	0.1588
CSF Glu, mmol/L	3.6 (3.2–8.6), 20	3.7 (2.5–5.2), 21	0.7345
CSF IgG, mg/L	38.1 (11.6–520), 18	64.4 (12–169), 17	0.0773
IgG Index, ×1000	503.7 (161.9–721.5), 11	636.2 (231.1–791.9), 6	0.2196
Qalb, ×1000	7.5 (2.6–70), 15	12.4 (2.2–63.8), 14	0.2172
Neuron-specific endase, μg/L	12.4 (7–47.9), 22	14.4 (8.7–180.5), 25	0.1146
AFP, ng/mL	2.4 (0.6–10.3), 28	2.6 (0.9–8.5), 26	0.6841
Carcinoembryonic antigen, μg/L	2.2 (0.5–8.1), 29	2.5 (0.5–13.1), 27	0.5550
TGAB, IU/mL	0.3 (0–175), 26	0.2 (0–38.7), 24	0.9530
TPO, IU/mL	0.7 (0.1–131), 26	0.6 (0.1–1,087), 24	0.8760
Other autoantibodies	10 (52.6%), 19	8 (44.4%), 18	0.7460
CK-MB, U/L	10 (4–341), 19	11 (4–98), 25	0.4610
AST/ALT, ×100	121.7 (32.5–666.7), 35	105.9 (37–650), 35	0.9111
BUN/Cr, ×100	6.9 (1.8–14), 35	6.3 (3–14.5), 35	0.9906
WBC, >9.5×10^9^/L	8 (22.9%), 35	10 (28.6%), 35	0.7845
PMN, >75%	11 (31.4%), 35	13 (37.1%), 35	0.8012
Platelet, <125×10^9^/L	1 (2.9%), 35	2 (5.7%), 35	1
Electroencephalogram	12 (70.6%), 17	9 (90.0%), 10	0.3630
Electromyography	15 (88.2%), 17	19 (90.5%), 21	1
Magnetic resonance imaging FLAIR/T2 Abnormalities	10 (33.3%), 30	8 (26.7%), 30	0.7782
Computed tomography finding on tumor	5 (18.5%), 27	8 (30.8%), 26	0.4734
Paraneoplastic neurological disorders vs autoimmune encephalitis	14 (60.9%), 23	24 (82.8%), 29	0.1463

*Data presented as positive specimen (%) sample size, or median (range) sample size*.

Multivariate regression analysis was then performed for all patients. Urinary incontinence/retention (*p* = 0.0388) and an immunocompromised state (*p* = 0.0247) were significant factors associated with a poor outcome. Nevertheless, duration prior to the hospital was not statistically associated with a poor outcome (*p* = 0.0674) (Table 5).

**Table 5:** Poor outcome factors in onconeural antibody associated disorders patients: multivariate analysis.

	OR (95% CI)	*p*-Value
Urinary incontinence/retention	27.22 (1.51–1,137.04)	0.0388
Immunocompromised state	34.6 (2.21–1,437)	0.0247
Duration prior to the hospital, d	1.01 (1–1.03)	0.0674
CSF Cl, mmol/L	0.91 (0.71–1.11)	0.3555
CSF IgG, mg/L	1 (0.98–1.01)	0.8512
CSF WBC, ×10^6^/L	1.1 (1.02–1.3)	0.1846

*CSF = cerebrospinal fluid, OR = odds ratio*.

## Discussion

In this study, we integrated PNDs and AE as a category named OAAD for the similarity of underlying pathogenesis and clinical management as follows. Most cases of OAAD are probably immune mediated, as demonstrated by the fact that anti-neuronal antibodies can be found in the CSF and serum (4). For paraneoplastic disorders, the ectopic expression of neuronal antigens within the tumor appears to contribute to breaking the immune tolerance and activating the immune response. For non-paraneoplastic disorders, molecular mimicry may initiate an immune response against neuronal antigens during the process of virus infection (10). The manifestation of OAAD can appear as encephalitis, encephalopathy, peripheral/autonomic neuropathy, or syndromes involving the neuromuscular junction. The clinical approach to diagnosing OAAD is to identify the neurological syndrome, detect specific antibodies in the serum or CSF, and verify the underlying cancer in some cases. In terms of clinical management, immunotherapy and tumor removal are the primary course of treatment.

Our study investigated the clinical presentation and management, CSF analysis, serum autoantibodies and tumor markers, the function of crucial organs, electrophysiology, and radiological findings in patients with OAAD. Furthermore, we used these data to identify potential predictors of outcome. In univariate analysis, duration prior to the hospital (*p* = 0.0224) and urinary incontinence/retention (*p* = 0.0043) were associated with a poor outcome. Furthermore, an immunocompromised state (*p* = 0.0944), the total number of tumors (*p* = 0.0542) and CSF IgG concentration (*p* = 0.0773) may also be associated with outcome. After multivariate regression analysis, urinary incontinence/retention and an immunocompromised state were shown to be significantly related to a poor outcome.

Urinary incontinence/retention is the main manifestation of damage to the autonomic nerve and its nerve center, including the lobulus paracentralis, hypothalamus, and parasympathetic center in the spinal cord. Our findings verified that this was the presentation of a serious injury to the nervous center and indicated a poor outcome. These results have not been reported previously for PNDs and AE. Further research is now required to explore the underlying pathogenesis of this condition. An immunocompromised state was confirmed as a factor associated with a poor outcome, as in a previous study of encephalitis (11). However, in the previous study of anti-NMDAR encephalitis, the predictors associated with a good outcome included early treatment, the lack of ICU requirement and a longer follow-up period (12), and therefore did not incorporate the factors utilized in the present study. More clinical research is now needed to identify the specific factors associated with outcome for patients with OAAD.

In terms of clinical presentation, we selected cognitive damage, mental and behavioral abnormality, confusion, epilepsy, focal deficits, urinary incontinence/retention, and temperature upon admission as influential factors with which to investigate outcome. Univariate and multivariate regression analysis confirmed that urinary incontinence/retention was a significant factor associated with poor outcome. Therapeutic measures, the total number of tumors and the duration prior to the hospital or during the hospital stay were also evaluated in the present study but did not show any significant relationship with outcome. Furthermore, ICU admission and mechanical ventilation did not show any relationship with outcome, which contradicted the results of a previous report (12). Univariate analysis showed that duration prior to the hospital was significantly associated with poor outcome (*p* = 0.0224), although this relationship was not significant when tested by multivariate regression analysis (*p* = 0.0674). In our cohort, duration prior to the hospital ranged from 1 week to 3 years. A longer time before the hospital visit always related to the progression of a potential tumor, the functional decline of major organs and a delay in the administration of appropriate treatments. This factor was verified as an indicator of poor outcome in the cohort study reported by Maarten (12).

In our CSF analysis, as well as investigating CSF components such as WBC, protein, Glu, and IgG, we also calculated the albumin quotient (Qalb) as a marker of the integrity of the blood–brain barrier, and IgG index as an estimate of intrathecal IgG synthesis (Tables 3 and 4). Univariate analysis showed that only CSF IgG may be associated with a poor outcome, although there was no statistical significance when tested by multivariate regression analysis. High IgG index, pleocytosis and increased protein levels usually appear within the first few days after neurological symptoms (4, 13–15). The dynamic changes of CSF components may be one of the factors associated with the lack of statistical significance in our present study, as might the low numbers of CSF tested.

In the present study, we also investigated tumor markers, onconeural antibodies and coexistent anti-thyroid autoantibodies, anti-nuclear/myositis/ganglioside antibody profiles, and anti-MPO/PR3/AchR/AQP4 antibodies. Our testing showed that 16/47 patients (34%) were positive for tumor markers and NSE, and that the next highest positive marker was CEA (9/56 patients, 16.1%). There were 51/70 patients (72.9%) who possessed onconeural antibodies, including Hu (13 patients), NMDAR (13 patients), and Yo (11 patients) (see Table 1). A total of 25 patients (35.7%) tested positive for serum autoantibodies. The coexistent autoantibody mainly included antithyroglobulin antibody (TGAb) (13 patients), anti-thyroid peroxidase antibody (TPOAb) (5 patients), ANA (5 patients). Neither univariate nor multivariate regression analysis revealed any significant relationship between antibody level and poor outcome. A previous study evaluated the coexistence of autoantibodies in cases of myasthenia gravis; 52% (39/75) patients tested positive for autoantibodies, including 27 cases with TPOAb, 17 cases with TGAb, and 17 cases of ANA (16). The pathogenesis underlying the coexistence of autoantibodies in the same patient remains unclear. Gene phenotypes, environmental factors, infection, immune dysfunction, and other factors may be connected with this phenomenon. The relationship between autoantibodies and the potential risks of autoimmune diseases, however, remains uncertain. Whether and how coexistent autoantibodies influence the therapeutic approaches and the prognosis of OAAD requires further research.

Clearly, the failure of crucial organs may be related to a poor outcome, at least to some extent. In the present study, we evaluated the function of important organs including heart, liver, kidney, and the hemopoiesis system. We tested AST/ALT, BUN/Cr, leukocytes, PMN, and thrombocytes in all patients, and CK-MB in 44 patients, in order to assess organic function. The number of patients with leukocytosis (WBC > 9.5 × 10^9^/L) was 10 (28.6%) in the group of poor outcome and 8 (22.9%) in the group of good outcome; for patients with thrombocytopenia (Platelets < 125 × 10^9^/L), the number was 1 (2.9%) and 2 (5.7%); for patients with PMN ratio rise (>75%), the number was 11 (31.4%) and 13 (37.1%), respectively. Other indicators of organ function were rarely abnormal in our cohort. All results were analyzed by both univariate and multivariate regression analysis but none of these tests revealed a relationship with poor outcome. The function of the main organs is seldom evaluated as an indicator of outcome in a cohort study. While we obtained a negative result in this study, we plan to perfect the protocol and increase sample size in order to create a more robust study.

Our present study also considered electrophysiology and radiological findings in our diagnosis and in the prediction of outcome. The number of patients with abnormalities in EEG was 12 (70.6%) in the good outcome group and 9 (90%) in the poor outcome group; for patients with abnormalities in EMG, the number was 15 (88.2%) and 19 (90.5%); for MRI FLAIR/T2, the number was 10 (33.3%) and 8 (26.7%); for CT finding on tumor, the number was 5 (18.5%) and 8 (30.8%), respectively (Table 4). The detailed data in the PNDs, AE, and possible OAAD groups are presented in Table 3. Further statistical analysis failed to reveal any significant differences in terms of indicators of outcome. As reported previously, few patients with anti-NMDAR encephalitis showed FLAIR/T2 abnormalities in the cerebral parenchymal (12–15). In another study, abnormalities were observed in brain MRI and EEG in 33 and 90% of all patients, respectively (12), which concurred with our own cohort.

Overall, there was a 15.7% mortality rate (11/70 patients) in our current study, which was higher than the rates of 9.5 and 9% reported in previous studies (11, 12). The number of dead patients distributed in PNDs was 54.5% (6/11), in AE 18.2% (2/11), in possible OAAD 27.3% (3/11). In the PNDs group, 24 patients (63.2%, 24/38) had a poor outcome; this compared to 35.7% (5/14) and 33.3% (6/18) in the AE and possible OAAD groups. In terms of treatment, there were 35 patients who were administered with immunotherapy, predominantly steroids or IVIg, or both. Of the patients receiving treatments, 19 patients were classified as having a good outcome while 16 were classified as having a poor outcome; univariate analysis showed that there was no difference between these two groups of patients (*p* = 0.4621).

The present study strengthens our holistic evaluation of crucial organs and analyzes the coexistence of antoantibodies and tumor markers in patients with OAAD, while also considering clinical presentation and management, CSF analysis, electrophysiology, and radiological findings. The present study was limited in several ways. First, there was a shortage of exhaustive and complete data. Second, the study was retrospective in design, which led to the shortage of data. Finally, due to the long period of time before hospital attendance (mean: 146.4 days; range: 7–1090 days), the pathophysiological status of patients changed and appropriate treatments, including tumor surgery and immunotherapy, were delayed. Collectively, these factors would have influenced the clinical presentation, radiological findings, and test results, and may also have influenced the outcome, at least to some extent.

## Conclusion

In the present study, 15.7% of our patients died and 50% of our patient cohort experienced a good or poor outcome, respectively. The distribution of mortality and outcome was not significantly different when compared across the PNDs, AE, and possible OAAD groups. In terms of indicators of prognosis, we identified that urinary incontinence/retention and an immunocompromised state represent significant factors that are associated with outcome (OR = 27.22 and 34.6, respectively) and portend a worse outcome.

## Ethics Statement

This study was carried out in accordance with the recommendations of the ethics committee of General Hospital of Jinan Military Command with written informed consent from all subjects. All subjects gave written informed consent in accordance with the Declaration of Helsinki. The protocol was approved by the ethics committee of General Hospital of Jinan Military Command.

## Author Contributions

BC and SL designed the study. SL and YQ contributed equally to this work and should be considered co-first authors. They analyzed the data and performed the writting of the manuscript. HH, HG, XW, and SM collected and sorted the clinical data. CL implemented laboratory technique and checked the testing result. BN accomplished the data statistics.

## Conflict of Interest Statement

The authors declare that the research was conducted in the absence of any commercial or financial relationships that could be construed as a potential conflict of interest.
