# Corrigendum: Clinical Characteristics and Predictors of Outcome for Onconeural Antibody-Associated Disorders: A Retrospective Analysis

**DOI:** 10.3389/fneur.2018.00729

**Published:** 2018-09-05

**Authors:** Shaohua Liao, Ying Qian, Huaiqiang Hu, Bing Niu, Hongwei Guo, Xiaoling Wang, Shuai Miao, Chuanfen Li, Bingzhen Cao

**Affiliations:** ^1^Department of Neurology, Graduate School of the Second Military Medical University, Shanghai, China; ^2^Department of Neurology, General Hospital of Jinan Military Command of PLA, Jinan, China; ^3^Department of Neurology, People's Liberation Army 152 Hospital, Pingdingshan, China

**Keywords:** onconeural antibody, clinical characteristics, management, outcome, predictors

In the original article, there was a mistake in the third last patient in table 1 is listed to have the onconeuronal antibody “Am”, which is corrected as “Amphiphysin”. The authors apologize for this error and state that this does not change the scientific conclusions of the article in any way.

In addition, there was an error in the conclusion of the abstract, caused by a misunderstanding during the language translation. The original text is:

By contrast, cerebrospinal fluid analysis showed that serum autoantibodies and tumor markers, the function of crucial organs, electrophysiology, and radiological findings were not associated with a poor outcome.

A correction has been made to the corresponding text in the conclusion of the abstract:

By contrast, the results showed that cerebrospinal fluid analysis, serum autoantibodies and tumor markers, the function of crucial organs, electrophysiology, and radiological findings were not associated with a poor outcome.

The authors apologize for this error and state that this does not change the scientific conclusions of the article in any way.

## Conflict of interest statement

The authors declare that the research was conducted in the absence of any commercial or financial relationships that could be construed as a potential conflict of interest.

